# Combating Scientific Misconduct: The Role of Focused Workshops in Changing Attitudes Towards Plagiarism

**DOI:** 10.7759/cureus.2698

**Published:** 2018-05-28

**Authors:** Farooq A Rathore, Noor E Fatima, Fareeha Farooq, Sahibzada N Mansoor

**Affiliations:** 1 Department of Rehabilitation Medicine, PNS Shifa Hospital, DHA II, Karachi 75500, Pakistan; 2 Department of Medicine, CMH Lahore Medical College and Institute of Dentistry, Shami Road, Lahore Cantt.; 3 Department of Biochemistry, Sir Syed Medical College for Girls, Karachi; 4 Department of Rehabilitation Medicine, Combined Military Hospital, Panoaqil, Pakistan

**Keywords:** plagiarism, pakistan, medical writing, knowledge and attitude, structured questionnaire, questionnaires, lmic, research misconduct, evaluation, teaching evaluation

## Abstract

Introduction

Scientific misconduct is a global issue. There is low awareness among health professionals regarding plagiarism, particularly in developing countries, including Pakistan. There is no formal training in the ethical conduct of research or writing for under- and post-graduate students in the majority of medical schools in Pakistan. Internet access to published literature has made plagiarism easy. The aim of this study was to document the effectiveness of focused workshops on reducing scientific misconduct as measured using a modified version of the attitude towards plagiarism questionnaire (ATPQ) assessment tool.

Materials and methods

A cross-sectional study was conducted with participants of workshops on scientific misconduct. Demographic data were recorded. A modified ATPQ was used as a pre- and post-test for workshop participants. Data were entered in SPSS v20 (IBM< Armonk, NY, US). Frequencies and descriptive statistics were analyzed. An independent sample t-test was run to analyze differences in mean scores on pre-workshop ATPQ and differences in mean scores on post-test scores.

Results

There were 38 males and 42 females (mean age: 26.2 years) who participated in the workshops and completed the pre- and post-assessments. Most (59; 73.75%) were final-year medical students. One-third (33.8%) of the respondents had neither attended workshops related to ethics in medical research nor published manuscripts in medical journals (32.5%). More than half (55%) admitted witnessing unethical practices in research. There was a significant improvement in attitudes toward plagiarism after attending the workshop (mean difference = 7.18 (6.2), t = 10.32, P < .001).

Conclusions

Focused workshops on how to detect and avoid scientific misconduct can help increase knowledge and improve attitudes towards plagiarism, as assessed by the modified ATPQ. Students, residents, and faculty members must be trained to conduct ethical medical research and avoid all forms of scientific misconduct.

## Introduction

Plagiarism is ”the appropriation of another person’s ideas, processes, results, or words without giving appropriate credit” [1]. This issue has gained worldwide importance, as medical research and writing evolve. Scientific writing has become an essential skill for those in academia and is imperative for the professional growth and development of the field. The culture of “publish or perish” has forced many into the unethical practices of plagiarism [2]. Scientific misconduct and plagiarism are global issues, plaguing not only developing countries but also technologically advanced nations, and are on the rise [3-4]. This is likely due to an increased awareness of scientific misconduct and the development of better software to detect plagiarism. Formal training in understanding the ethical aspects of medical research and writing at the undergraduate or post-graduate level is lacking in most medical schools around the globe. This is of particular importance for developing countries where students and faculty usually do not have adequate access to scientific literature and strong library services. The three major misconducts in scientific research and writing are fabrication, falsification, and plagiarism [1]. Plagiarism can be compared to the proverbial hydra, as it also has many heads (types), from copying to paraphrasing, patchworking, poor or no citations/quotations, paying for getting articles written, and collusion with other students [5]. The accessible Internet has made plagiarism relatively easy [6].

There are established guidelines and codes of conduct regarding scientific misconduct and plagiarism, which have been adopted by medical journals and universities across the globe [7]. Still, many authors remain unaware and plagiarize.

The situation is no different in Pakistan. There is no defined curriculum or formal training for medical research and writing for the undergraduate medical students in the majority of medical schools in the country. Although faculty members are required to do medical writing and publish a certain number of manuscripts for promotion; they too are not formally trained to write for referenced biomedical literature. The revision of the faculty promotion rules by the Pakistan Medical and Dental Council (PMDC), globalization, the migration of physicians abroad, and international exposure has led to a paradigm shift in medical research and writing in Pakistan. These factors, combined with the current pressure to publish research, sometimes results in the author engaging in unethical practices to achieve professional goals. Even faculty members are often unclear about the implications of indulging in deliberate or unintentional plagiarism and are unable to guide their students and residents.

We have been conducting workshops on medical writing and mentoring students and peers since 2014 [8]. We observed a low level of awareness regarding plagiarism among the participants of these workshops. We conducted a cross-sectional survey to assess the knowledge and attitudes of the students and faculty towards plagiarism using the modified version of attitude towards plagiarism questionnaire (ATPQ) [9]. It revealed that the general attitudes of Pakistani medical faculty members and medical students towards plagiarism were positive. This has also been confirmed by other authors in Pakistan [10]. We aimed to address this issue by conducting a series of focused workshops on scientific misconduct and plagiarism for the students and faculty members.

The objective of our study was to assess the effectiveness of focused workshops on plagiarism as a tool for change in attitudes towards plagiarism as assessed by a modified version of ATPQ.

## Materials and methods

Ethics approval was obtained from the ethics review committee of CMH Lahore Medical College and Institute of Dentistry. We used a modified version of the ATPQ, which has been validated for use in Pakistan [9].

A total three workshops of three-hour duration each, titled “Scientific misconduct, plagiarism and ethical aspects of medical research and writing: What you need to know?”, were planned and facilitated by two authors (Rathore and Mansoor) between January and June 2016. Both are published researchers who have conducted more than 60 workshops on medical writing. The aims of the workshops were to provide an overview of the topic, describe different forms of scientific misconducts and plagiarism, relating to the local academic environment, and offer guidance on avoiding scientific misconduct and plagiarism. The workshops were facilitated in four sessions of approximately 40 minutes each. The workshop program details are described in Table 1.

**Table 1:** Details of the different components of the workshop COPE: Committee on Publications Ethics, JCPSP: Journal of College of Physicians and Surgeons of Pakistan, JPMA: Journal of Pakistan Medical Association, ICMJE: International Committee of Medical Journal Editors

Main Component	Details
Introduction to scientific misconduct; local and global perspectives	Standard definitions and types of scientific misconduct Overview of scientific misconduct and plagiarism in the published medical literature all around the globe Real-life examples of scientific misconduct and plagiarism from Pakistan Examples of punishments (including retractions) and public humiliations from COPE, JCPSP, JPMA, and Retraction Watch
Authorship criteria and unethical authorship	Value of authorship Discussion of the ICMJE criteria of authorship Different types of unethical authorships
Plagiarism, unethical publishing, and other issues	Different types of plagiarism Different types of unethical publishing Conflict of interest Copyright issues Informed consent
How to detect and avoid plagiarism?	Contributory factors towards plagiarism Expert advice to detect plagiarism Role of plagiarism-detection software Summary and take-home message

In case of medical students, they were nominated by the college administration based on their roll numbers. The participants in the workshop for the faculty were registered by the coordinator in that institute who gave an open call for participation. We did not exclude anyone who was interested in participating in these workshops. The participants were encouraged to ask questions and clarify any ambiguities during the sessions as well as at the end of each presentation. Two hands-on exercises on authorship and unethical practices were conducted and the answers were discussed and debated in group discussion.

At the start of the workshops, participants were briefed about the ATPQ and the need to objectively measure the attitudes towards plagiarism and the impact of the workshops. The questionnaire had three parts. The first part was informed consent, which assured the participants of anonymity and explained the rationale of the study. The second part was demographic data, including information if the respondent had attended a similar, focused workshop on plagiarism or had witnessed any unethical practice. The third part was the ATPQ. The ATPQ consisted of 22 questions with three options (agree, neutral, disagree). The participants had 10 minutes to complete the questionnaire, which was then collected by one of the authors. After the pre-workshop ATPQ assessment, the three-hour workshop was conducted. To evaluate the improvement in attitudes towards plagiarism, respondents were tested again using the same ATPQ after the workshop.

Data were entered in IBM SPSS Statistics for Windows, Version 20.0 (IBM Corp., Armonk, NY, US). Frequencies and descriptive statistics were run for demographics and respondents’ characteristics. An independent sample t-test was run to analyze differences in mean scores on pre-workshop ATPQ and dichotomous (Yes/No) variables.

To evaluate the improvement in attitudes towards plagiarism, a t-test for dependent samples was run to analyze differences in mean scores on ATPQ prior to and after the workshop was delivered. Prior to running this test, Kolmogorov-Smirnov and Shapiro-Wilk tests of normality were run to assess the assumption of normality for differences in ATPQ scores prior to and after the workshop.

## Results

There were 38 males and 42 female participants with a mean age of 26.28 (+6.7) years. The majority were final year medical students 59 (73.75%) and 21 (26.25%) were faculty members. One-third (33.8%) of the respondents had never attended workshops, seminars, or lectures related to ethics in medical research and writing before this workshop, and only 32.5% had published manuscripts in peer-reviewed medical journals. Most of the respondents (47/80) did not have a research supervisor or mentor. More than half (55%) indicated they had witnessed unethical practice or scientific misconduct among their colleagues and seniors (Table 2).

**Table 2:** Responses of respondents toward questions and differences on mean ATPQ scores ATPQ: attitude towards plagiarism questionnaire

Question	Response	Frequency (%)	Mean Score on ATPQ	t-value	P-value
Have you attended workshops, seminars, or lectures related to medical writing before this workshop?	Yes	27 (33.8%)	40.37 (7.1)	-0.29	0.77
	No	53 (66.2%)	40.84 (6.4)		
Have you been involved in medical research and writing before (thesis, dissertation, or manuscript writing)?	Yes	42 (52.5%)	38.68 (6.01)	-2.9	0.004
	No	38 (47.5%)	42.89 (6.7)		
Have you published manuscripts in peer-reviewed medical journals? (includes original research, case reports, brief reports, and special communications)	Yes	26 (32.5%)	39.10 (6.4)	-1.5	0.14
	No	54 (67.5%)	41.44 (6.7)		
Do you have a supervisor/mentor/instructor in medical research and writing?	Yes	33 (41.3%)	38.30 (6.2)	-2.8	0.007
	No	47 (58.8%)	42.35 (6.5)		
Have you witnessed any unethical practice or scientific misconduct among your colleagues and seniors?	Yes	44 (55%)	40.24 (6.2)	-0.72	0.47
	No	46 (45%)	41.34 (7.2)		

A t-test for paired samples was run to analyze the difference in mean scores on ATPQ reported prior to and after the workshop. The respondents reported a significant improvement in attitudes toward plagiarism after the delivery of workshop (mean difference = 7.18 (6.2), t = 10.32, P < .001). In addition, respondents who had a mentor/supervisor and had previous experience in medical writing had a significantly less positive attitude toward plagiarism. Those who had attended workshops and seminars related to medical writing before the present workshop did not show any significant difference in mean scores on pre-workshop ATPQ than their counterparts.

## Discussion

This study demonstrates that the majority of students and faculty members in Pakistan do not receive formal training in research and scientific writing misconduct, including plagiarism. This is consistent with a recent report that showed a lack of knowledge of scientific misconduct among medical students from public and private medical colleges in Karachi [11]. In addition, participants in these workshops generally lacked the skills and expertise to detect and avoid scientific misconduct or plagiarism. Many participants (55%) had witnessed unethical practices related to research and writing at their workplace. We observed that such focused workshops can enhance the understanding of students and faculty members of plagiarism and other forms of unethical practices in research and writing.

One of the major reasons for not recognizing plagiarism in Pakistan is probably related to basic education before medical college. In the traditional educational system of Pakistan, the verbatim reproduction of content from books is considered a normal practice and no referencing is required [12-13]. Most students in medical schools and at the post-graduate level follow the same practice until they are corrected by their teachers or are caught unaware during the submission of their assignments or research manuscripts. In 2014, Ghias et al. reported that a formal ethics curriculum in public medical schools is lacking [14]. Another important finding was that students did not refrain from engaging in scientific misconduct even when they were able to identify the academic misconduct.

Our study revealed a lack of awareness about plagiarism, with significant improvements in knowledge following a focused workshop on plagiarism. This is similar to Vuckovic et al. from Serbia who demonstrated that even a short course in science ethics can have a great impact on the attendees and enhance their knowledge of the responsible conduct of research. Such interventions can also change behaviors regarding the reluctance to react publicly and punish wrongdoers [15]. Kirsch et al. conducted a series of plagiarism awareness workshops at the University of South Carolina, USA, and concluded that a structured system of workshops about scientific misconduct should be arranged [16]. The same researchers also suggested that librarians can play a role in conducting online workshops for faculty and students located in distant campuses. In Pakistani medical schools, well-equipped medical libraries are rare and the majority of librarians are not trained to provide guidance to students and faculty regarding scientific and medical writing misconduct [17-18].

Another important finding in our study was the lack of knowledge of authorship criteria. None of the participants in these and previous workshops were aware or clear about the globally accepted authorship criteria of the International Committee of Medical Journal Editors( ICMJE) [19]. This is a major issue in Pakistan, with undergraduate medical students and postgraduate residents working with a senior faculty member. It is not uncommon for the faculty member or head of the department to demand first authorship without any significant contribution. We dedicated one section of the workshop on this topic and conducted one exercise to reinforce the concept. Knowledge of authorship criteria can help authors know their rights as well as contributions towards hierarchy in the authorship of a particular manuscript and avoid and contest unethical encroachments [20].

Based on the authors' personal experiences and feedback and discussion with the workshop participants, certain unethical issues were highlighted during these workshops. Most of them have not formally been documented or reported in Pakistan. It is a common misconception in Pakistan that it is ethically correct to split a single dissertation or thesis into multiple studies in order to increase the number of publications. We noticed that the participants were not aware or clear about “salami” publications and how data slicing constitutes an ethical concern [21]. We clearly explained this concept, elaborated it with examples, and discussed the drawbacks of creating multiple manuscripts from a single research project. We have also noticed that the major issue in Pakistan is lack of training. This has been demonstrated in a previous survey in three medical institutes in Karachi, Pakistan, which found that the major cause of plagiarism was lack of training in research methodology and referencing techniques rather than malicious intent in most the cases [10].

We recommend the following measures to combat the rising menace of scientific misconduct and plagiarism in Pakistan and other countries:

a) All stakeholders of under and postgraduate medical education in Pakistan, including PMDC, College of Physicians and Surgeons of Pakistan (CPSP), and Higher Education Commission (HEC), along with the Ministry of Health and Education, should devise national guidelines regarding scientific misconduct and plagiarism. These should be uniformly implemented all across the country.

b) A formal training program and a national curriculum on scientific misconduct and research ethics for undergraduate and postgraduate studies must be developed. This should be a combination of lectures, seminars, and training workshops.

c) There is a need to scrutinize and train supervisors, as they are directly responsible for the training of future generations of researchers, trainers, and teachers. Many supervisors in Pakistan do not provide formal guidance to their students in research and writing and only sign the first page of a thesis without even reading the whole text.

d) There should be zero tolerance towards all forms of scientific misconduct and plagiarism with a penalty imposed on the offenders even if they are senior faculty members [22].

e) The use of plagiarism-detecting software must be encouraged in all teaching institutions [10], as it is an effective tool to detect plagiarism [23].

f) Researchers must plan everything, including writing the manuscript in advance in order to avoid a last-minute rush to meet tight deadlines. A lack of planning leads to a last-minute panic, which can result in adopting unethical shortcuts.

g) Concerns have been raised about the institutional review boards/ethics review committee in Pakistan as being "rubber-stamping committees" [24]. A majority of the individuals working in these committees do not have the required training or cannot spare adequate time for the job. There is an urgent need to train individuals and to strengthen the institutional review boards/ethics review committee in every medical institute of the country.

h) There is a need to address the current culture of “publish and perish” in Pakistan, which has led to a rat race of publishing low-quality manuscripts just for the sake of promotions. The focus must change from quantity to quality [25].

i) A central registry of researchers and authors should be created similar to the AuthorAID mentor program [26]. This will allow potential supervisors to offer their services and make it possible for young researchers to identify a suitable mentor for their research journey.

j) Whistle-blowing is now considered an ethical activity in the developed world, as it has the potential to identify wrongdoings in the healthcare sector [27-28]. It is time that Pakistani academia also adopts this global norm and starts recognizing the value of whistle-blowers, as they can help highlight unethical research and scientific misconduct, which otherwise goes unnoticed. They should be provided legal cover and guarded against exploitation when they expose a wrongdoing.

Limitations

This study has some limitations that warrant mention. The sample size was limited to 80 participants, which is very small considering that, currently, there are more than 140 medical and dental colleges in Pakistan with thousands of students and faculty members. For a majority of the students, this was the first workshop on scientific misconduct and some of them complained of information overload in a single workshop, as many concepts were new for them and difficult to understand in a single sitting. We did not document the long-term outcomes of this training on the research output of the participants and if it actually resulted in a sustained change in attitude towards plagiarism.

## Conclusions

The attitude towards plagiarism and the knowledge of scientific misconduct was poor among our workshop participants. This is likely due to low awareness and the absence of scientific misconduct in curriculum both at the under- and postgraduate levels. Having a research mentor and prior experience in medical writing is associated with a better awareness of plagiarism. Awareness of plagiarism and scientific misconduct can be significantly increased with focused workshops on plagiarism.

## Appendices

**Table 3:** Attitude toward plagiarism questionnaire (22 items, validated in Pakistan) Scoring: It has a three-point Likert scale response pattern: agree (coded as 3), neutral (coded as 2), and disagree (coded as 1). The total score is the sum of all the 22 items. There is no negative scoring. Increasing scores reveal a higher tendency toward plagiarism.

Statement	Agree	Neutral	Disagree
1. Since plagiarism is taking other people's words rather than tangible assets; it should NOT be considered a serious offense.			
2. It is justified to use previous descriptions of a method because the method itself remains the same.			
3. Self-plagiarism is not punishable because it is not harmful (one cannot steal from oneself).			
4. Plagiarized parts of a paper may be ignored if the paper is of great scientific value.			
5. Self-plagiarism should not be punishable in the same way as plagiarism is.			
6. Young researchers who are just learning the ropes should receive milder punishments for plagiarism.			
7. I could not write a scientific paper without plagiarizing.			
8. Short deadlines give me the right to plagiarize a bit.			
9. It is justified to use one's own previously published work without providing a citation in order to complete the current work.			
10. Authors say they do NOT plagiarize, when, in fact, they do.			
11. A plagiarized paper does no harm to science.			
12. Sometimes, one cannot avoid using other people's words without citing the source because there are only so many ways to describe something.			
13. If a colleague of mine allows me to copy from her/his paper, I'm NOT doing anything bad because I have his/her permission.			
14. Those who say they have never plagiarized are lying.			
15. Sometimes, I'm tempted to plagiarize because everyone else is doing it (students, researchers, physicians).			
16. I keep plagiarizing because I haven't been caught yet.			
17. I work (study) in a plagiarism-free environment.			
18. Plagiarism is not a big deal.			
19. Sometimes, I copy a sentence or two just to become inspired for further writing.			
20. I don’t feel guilty for copying verbatim a sentence or two from my previous papers.			
21. Plagiarism is justified if I currently have more important obligations or tasks to do.			
22. Sometimes, it is necessary to plagiarize.			
